# An activity-based synthetic population of Gothenburg, Sweden: Dataset of residents in neighbourhoods

**DOI:** 10.1016/j.dib.2024.110945

**Published:** 2024-09-14

**Authors:** Sanjay Somanath, Liane Thuvander, Alexander Hollberg

**Affiliations:** Department of Architecture and Civil Engineering, Chalmers University of Technology, Sven Hultins gata 6, Göteborg 412 58, Sweden

**Keywords:** Mobility, Activity, Energy, Neighbourhood-planning, Demand-modelling, Accessibility, Equity

## Abstract

A synthetic population is a distribution of synthetic agents that replicates the demographic distribution of a real-world population based on census records. This paper presents an end-to-end model to generate a synthetic population of residents in Gothenburg, Sweden, along with activity schedules and mobility patterns for present and past populations. Using a stochastic modelling approach, we describe the model and present its corresponding dataset. The model is designed for applications in neighbourhood planning and includes detailed replicas of people in different neighbourhoods of Gothenburg organised as persons, households, houses, buildings, and daily activity chains. While the persons, households, and houses are synthetic replicas, they are connected to existing buildings. The model considers the allocation of primary and secondary locations based on a gravity model, realistic routing for active, public, and private motorised modes of transportation and allows users to introduce new buildings and amenities if needed. The model aims to impute national-level mobility patterns from a household travel survey and apply them locally to capture the nuances of a neighbourhood's built environment and demographic composition.

Specifications Table

**Table:** 

Subject	Data Science - Data Engineering
Specific subject area	Activity-based synthetic population modelling
Type of data	Raw data is provided in an SQL database with multiple linked tables for each neighbourhood.
Data collection	A synthetic population of residents within the primary area administrative boundaries of Gothenburg, Sweden, created from simulations in Python. The proposed model integrates primary datasets to generate synthetic individuals, households, and activity chains for each neighbourhood in Gothenburg.
Data source location	Primary sources include Statistics Sweden (SCB) Population data for Gothenburg, National Household Travel Survey (NHTS), The Swedish Cadastral Agency (Lantmäteriet) Building Footprints, OpenStreetMap Road Network and amenity locations, and City of Gothenburg data for schools and playgrounds.
Data accessibility	Repository name: Zenodo Data identification number: 10.5281/zenodo.10801936 Direct URL to data: www.doi.org/10.5281/zenodo.10801935 Instructions for accessing the data: Download the raw database files and open them using a SQLite3 database explorer.

## 1 Value of the Data

This dataset is generated using a synthetic population model for Gothenburg, Sweden, as described in this paper. The model combines local demographic and household data with data from a National Household Travel Survey (NHTS) using a combination of probabilistic assignment and Machine-Learning (ML) algorithms to create a spatially disaggregated representation of the city's population at the neighbourhood level. The model simulates individual and household travel patterns across different urban scenarios by incorporating a multi-dimensional representation of residents at a neighbourhood level and a multi-model routing pipeline. This dataset can serve multiple purposes across different domains:

• **Seed Population for Agent-Based Models:** Agent-based models require a high-quality synthetic population to establish the agents in the model. This dataset can be used to create synthetic agents to model interactions in various urban systems in Gothenburg at the neighbourhood level. The attributes of individuals and households can be supplemented with additional attributes to motivate the agents’ behaviour, hence saving modellers the need to generate their synthetic data.
• **Urban and Neighbourhood Planning:** This dataset offers valuable perspectives for neighbourhood planning, such as assessing the potential impacts of planning strategies on community liveability and urban sustainability. For example, the synthetic population and their activity schedules can provide neighbourhood planners with a spatially and temporally disaggregated amenity demand to plan the distribution of new amenities. Neighbourhood planners can also use the amenity engagement data to identify activities that could occur in the same location but at different times, hence improving the utilisation of the location and benefiting the neighbourhood.
• **Energy Demand Modelling:** This dataset enables exploration into how changes in urban form and population dynamics influence city-wide energy needs. It can support energy modellers in evaluating different energy policies and their potential effects on energy consumption. For example, the aggregated activity engagement data can provide modellers with data to assess which locations are occupied and at what hours on a neighbourhood level. This information can be used to size local energy systems optimally.
• **Behavioural Studies:** This dataset can be used to conduct behavioural studies at a neighbourhood level. Modelling human behaviour at the neighbourhood level often requires access to the microdata of residents, which can be costly or unavailable altogether. This dataset can allow researchers to develop their models using synthetic data in cases where real-world data is unavailable. It allows for the analysis of residents’ behaviour under different policy frameworks. It offers insights while maintaining individual privacy on energy consumption, appliance use behaviour, or the tendency to participate in different activities.
• **Digital Twinning and Urban Simulation:** A digital twin is a virtual representation of a physical system. An essential component in digital twins is integrating real-time sensor data like pedestrian flow or occupancy of spaces. In the development of urban digital twins, there is a need for real-time data. However, using real-time data from sensors can be challenging and costly. This dataset offers a stand-in for real-time data, enabling comprehensive simulations. Digital Twin developers can use the synthetic population to create placeholder IoT sensor data based on their modelling requirements.

## 2 Background

Most computational analysis methods in neighbourhood planning evaluate spatial accessibility through static accessibility metrics like space syntax [1] and walk score [2]. These static accessibility-based metrics do not evaluate equitable access in terms of socio-demographic characteristics or show the distributional impacts of neighbourhood planning on the residents. However, activity-based modelling and synthetic populations have long been used to model activity-travel patterns [3] and estimate urban energy demand from synthetic building stocks [4]. A synthetic population is a distribution of synthetic agents that replicates the demographic distribution of a real-world population based on census records.

We developed an activity-based synthetic population model and produced this dataset to help planners evaluate the social consequences of their neighbourhood designs and to help others interested in modelling neighbourhood-level consequences in Gothenburg, Sweden. Using a synthetic population rather than a real population offers several advantages particularly in terms of privacy, scalability and flexibility. Unlike real data, which may be restricted due to privacy concerns and cannot not capture the changes due to possible future, synthetic data is inherently anonymised and can be readily adapted. Synthetic populations can also be scaled to different geographical levels which can be challenging in the case of real data. Additionally, synthetic populations can be easily modified to reflect hypothetical scenarios with regards to changes in demographic, urban planning or policy.

While existing models of synthetic populations, such as SySMo by Tozlougolu et al. [3], exist for Sweden, our model differs from it in some significant ways. The SySMo model has a national scope intended to represent the entire population of Sweden and uses an Origin-Destination (OD) matrix derived from an external mobility demand model. Additionally, the routing of agents between the origin and destination is not currently within the scope of the SySMo model.

Our model is developed to be used at a neighbourhood level and is limited to the city of Gothenburg rather than having a national scope. Therefore, we use detailed demographic statistics at the neighbourhood level to achieve this. Second, our model uses destination locations and simulated routes taken by individual residents using a multi-modal transport network rather than OD matrices. Finally, in our model, we consider the routing of residents between their origins and destinations, accounting for their mode choices. While the two models share similar methods, they have different use cases and geographical extents.

The synthetic population model accepts, among other data sets, information on the location of amenities and residential buildings to evaluate the daily activity patterns of the residents in those buildings. The developed model allows planners to add or remove street segments, buildings or amenities to compare scenarios and regenerate the accompanying dataset.

## 3 Data Description

**Fig. 1:**
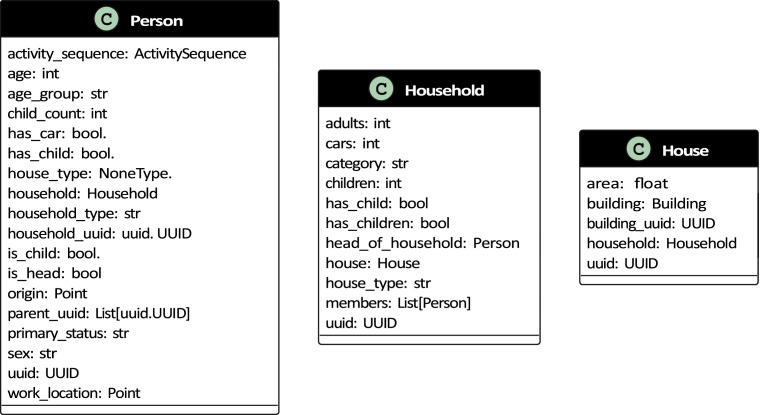
Class diagrams for person, household and house.

The synthetic population model generates different demographic objects that make up the synthetic population. These include a person, household, house (see Fig. 1 for a class diagram), building, activity and an activity sequence (see Fig. 3 for a class diagram) comprising individual activities.

### 3.1 Person

A *person* object consists of demographic attributes such as age, sex, household type, primary status (studying, working or inactive) and a unique identifier. Further, the person object is equipped with relational attributes such as a household identifier, the origin of the building they belong to, a parent identifier and a work location.

**Fig. 2:**
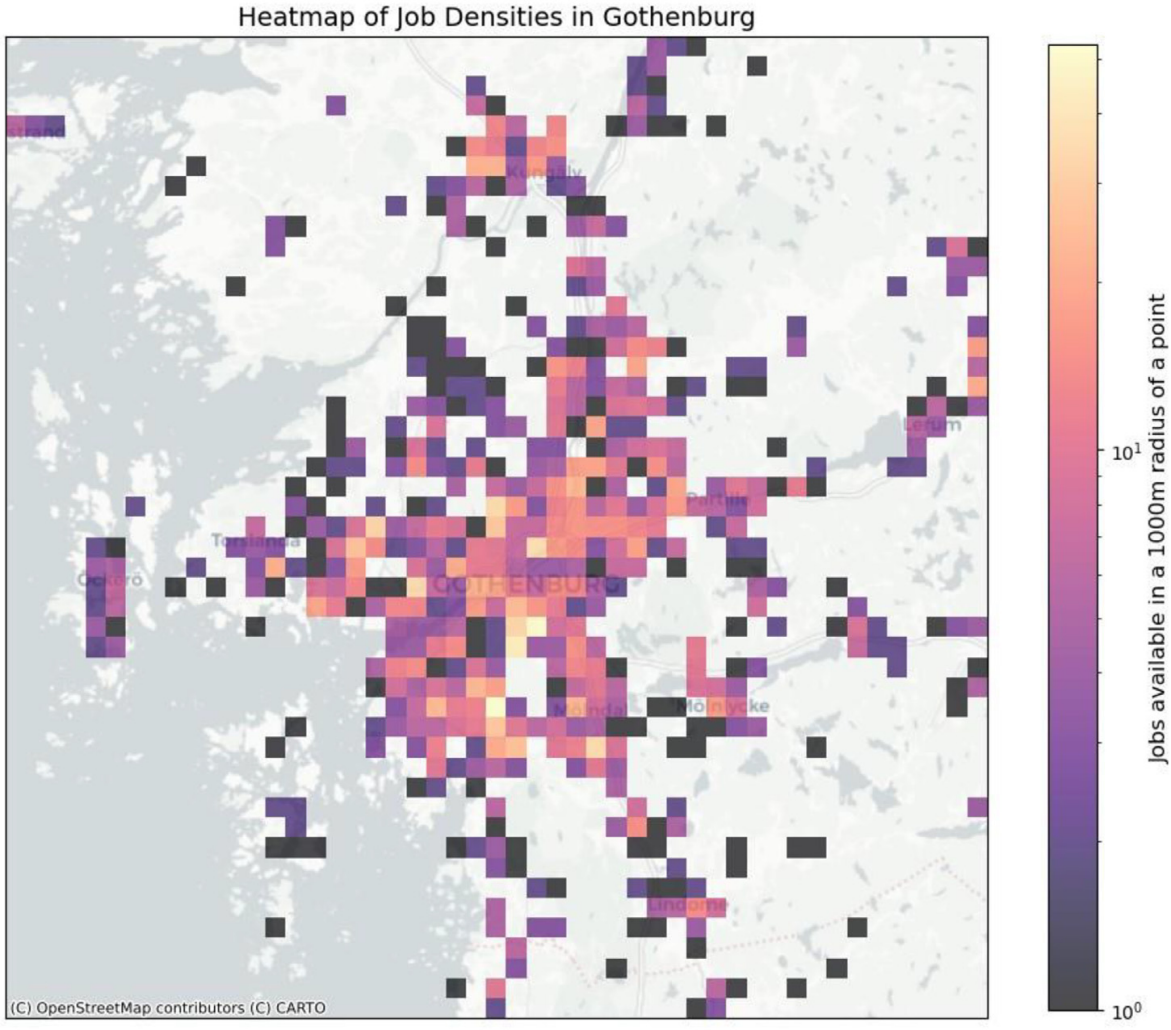
Job densities in Gothenburg.

The person object also references a work location based on pre-calculated job densities in Gothenburg (see Fig. 2) as a coordinate in the local projection system (EPSG 3006), car ownership, number of children and an *activity sequence* object.

### 3.2 Household

A *household* represents a collection of persons that live together. It comprises a household category, house type, a list of household members and a unique identifier. The household object contains relational attributes such as a reference to a house object and preferred destinations for different activities related to the household. Additional attributes include the number of cars in the household and the number of children in the household. The household class diagram is shown in Fig. 1.

### 3.3 House

Each household is assigned a *house* object that represents the housing unit that the residents occupy. A house is associated with a physical building in Gothenburg. For single-family houses, a single house object is related to a building; for multi-family houses, multiple house objects are associated with a Building. The house object contains attributes like floor area, reference to a building, reference to a household and a unique identifier. The house class diagram is shown in Fig. 1.

### 3.4 Building

**Fig. 3:**
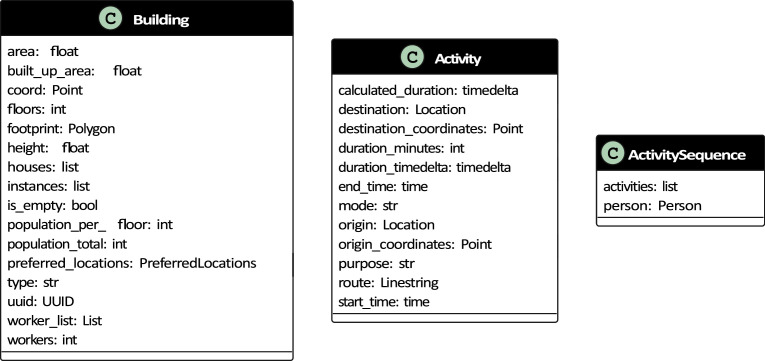
Class diagrams for building, activity and activity sequence.

A *building* object refers to an existing building in Gothenburg and references its physical location. The building object contains the footprint area, total built-up area, coordinates of the building (in EPSG:3006 coordinate reference system), the height of the building as calculated from the laser point-cloud data provided by Lantmäteriet [5], population per floor, total feasible population for the building and a unique identifier. Additionally, the building references a list of house objects contained within it, a list of all people living in the building. Fig. 3 shows the building class diagram.

### 3.5 Activity

**Fig. 4:**
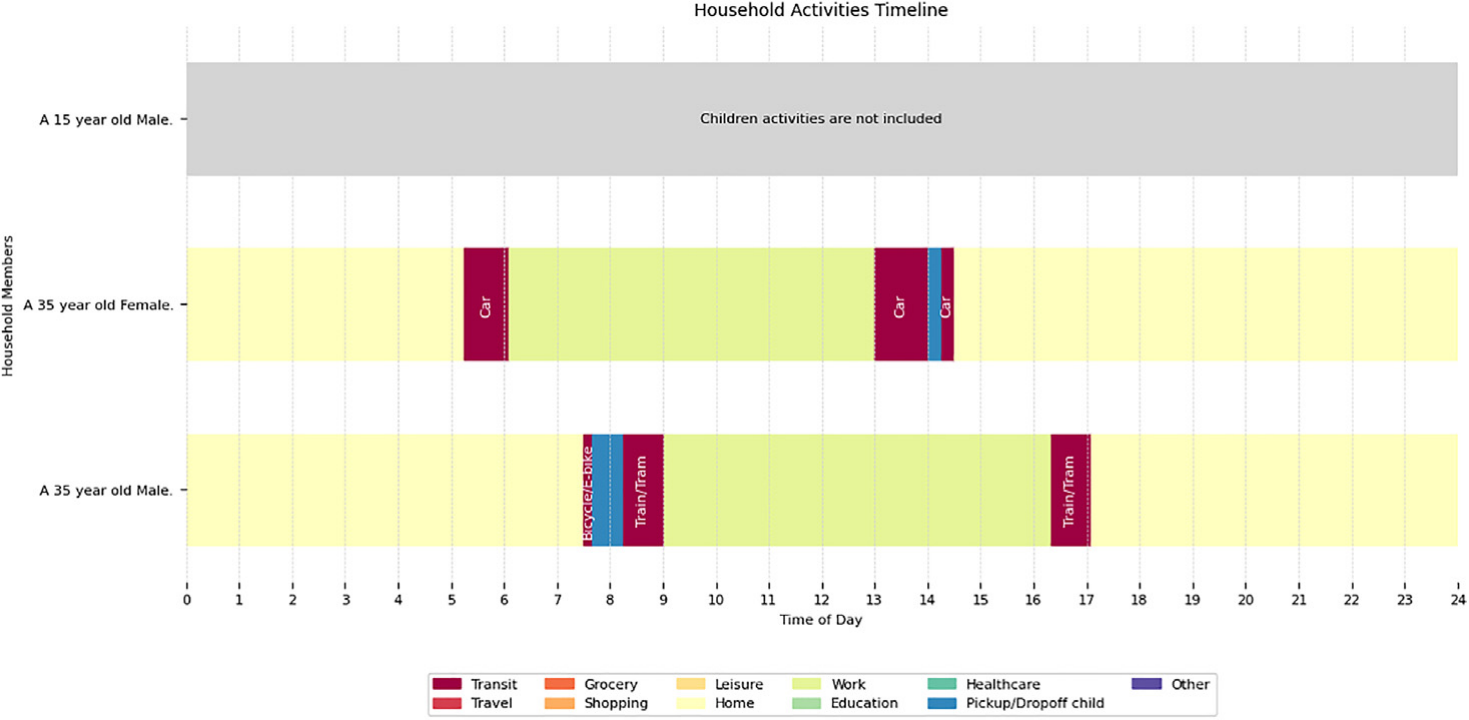
A sampled synthetic activity sequence.

An *activity* object consists of an activity purpose, start time, duration, end time and a mode of travel based on a sampled and matched activity sequence from the NHTS. Fig. 3 shows the activity class diagram. Fig. 4 shows the sequence of individual activities associated with a person.

### 3.6 Activity Sequence

The *activity sequence* object is a list of activity objects sampled from the NHTS. Each adult is assigned an activity sequence. Fig. 3 shows the activity sequence class diagram, and Fig. 4 shows a randomly sampled household and the activity sequences of the individual and their activity sequences.

**Fig. 5:**
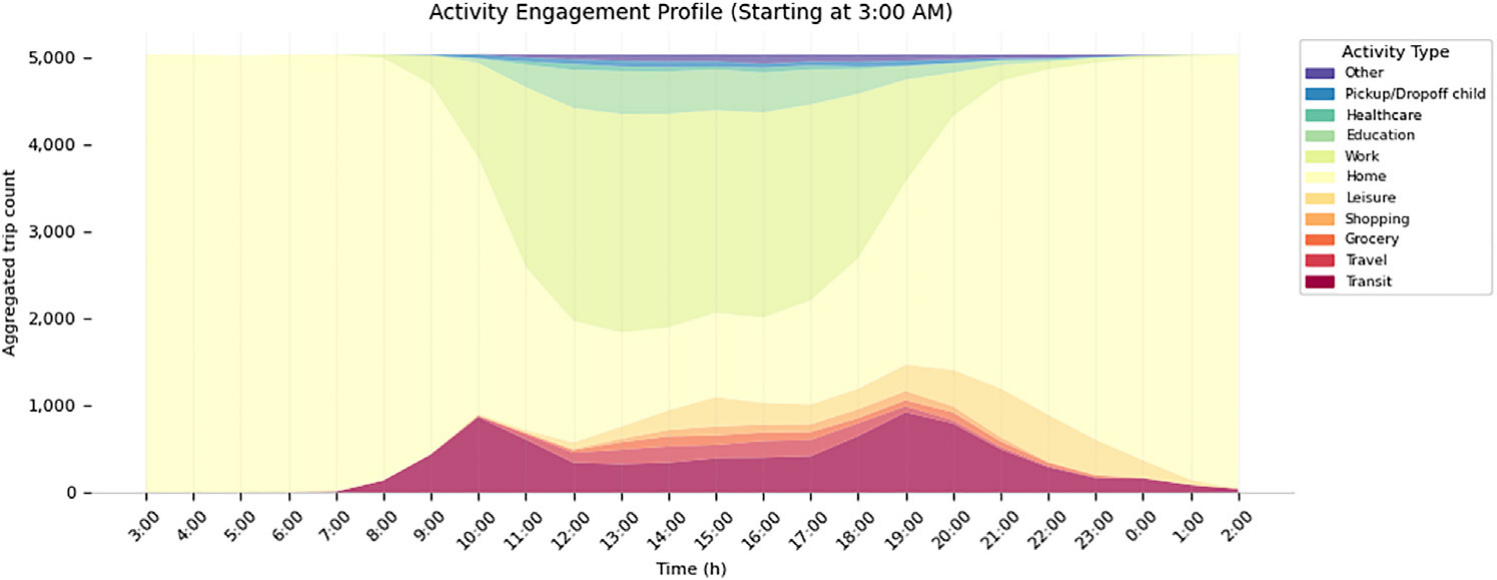
Aggregated activity engagement profile for the synthetic population based on sampled and matched activity sequences from the NHTS for neighbourhood Guldheden.

**Fig. 6:**
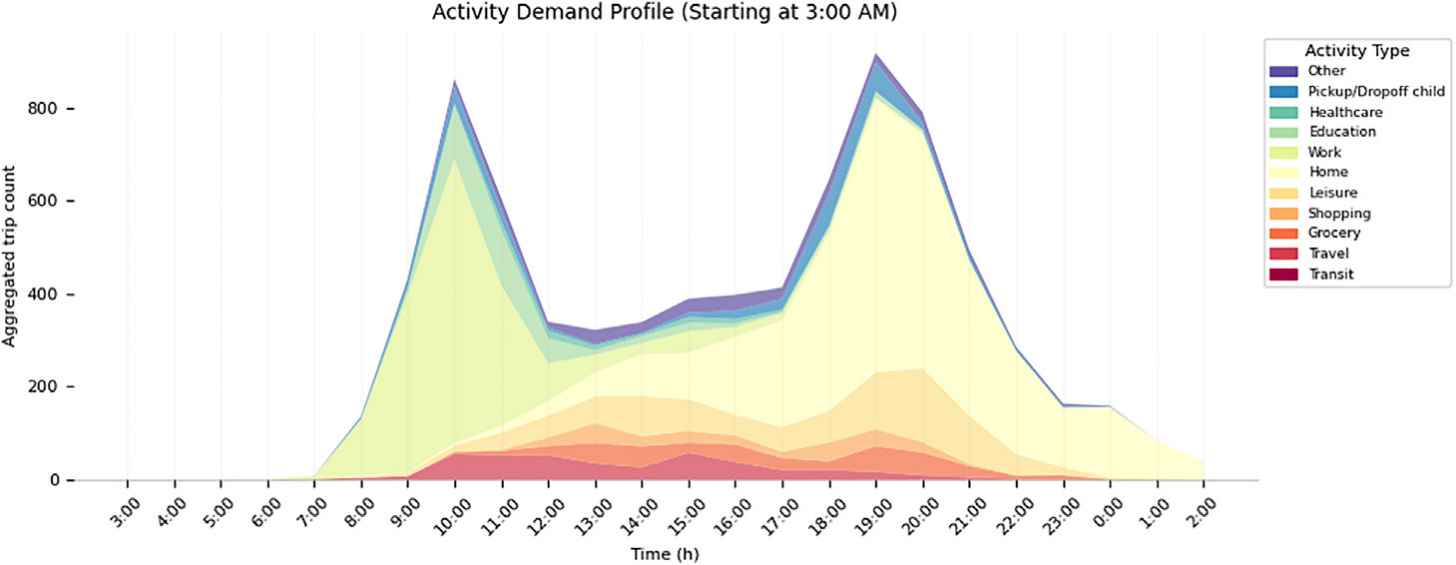
Aggregated activity demand profile for the synthetic population based on sampled and matched activity sequences from the NHTS for neighbourhood Guldheden.

Fig. 5, Fig. 6 show a temporally aggregated activity engagement profile and activity demand profile for a selected neighbourhood in the dataset.

## 4 Experimental Design, Materials and Methods

This section describes the data requirements for the synthetic population model and the data sources used to generate the synthetic population. The data requirements are listed in Table 1, and the data sources are described in detail in the following sections.

**Table 1:** Data requirements for the synthetic population model.

Data Requirement	Data Source
Household demographics	Göteborgs Stad [6]
Household location	Göteborgs Stad [7]
Household vehicle ownership	Göteborgs Stad [6]
School location	Göteborgs Stad [8]
Travel diary	Trafik Analys [9]
Building footprints	Lantmäteriet [5]
Street network	OpenStreetMap contributors [10]
Transit Feed (GTFS)	Trafiklab [11]

### 4.1 Swedish NHTS

A NHTS is a survey of the household travel patterns in a region. The Swedish NHTS is conducted annually and comprises around 35,000 respondents [9]. The survey includes questions about the household demographics and the travel patterns of the household members through a travel diary. The travel diary consists of a record of the travel patterns of the household members for 24 h. The travel diary consists of the following information, among others:

• The start and end times of the trip
• The mode of transport used for the trip.
• The purpose of the trip
• Household demographics include number of cars, children and household size.

### 4.2 Demographics

**Fig. 7:**
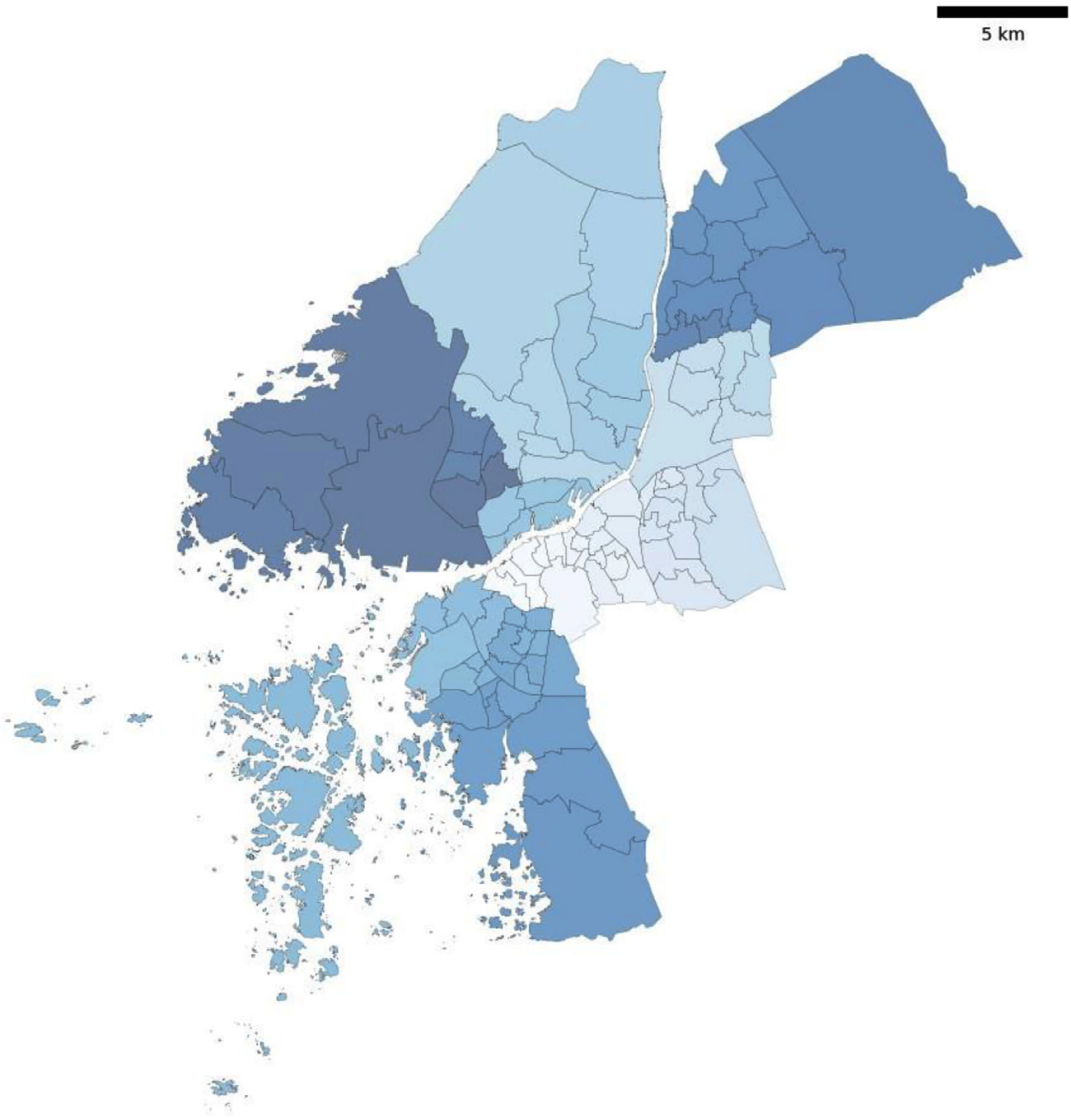
Primary areas of Gothenburg. The grouping of colours represents larger groupings of neighbourhoods into Nordost, Centrum, Sydväst, Hisingen and industrial areas.

The Swedish Department of Statistics (SCB) provides detailed demographic data at a high spatial resolution. For the city of Gothenburg, SCB offers a separate data portal, which includes demographic data at a primary area level [7], corresponding to a neighbourhood. The Gothenburg data portal makes this data available for all years, from 2003 to the present. There are 96 primary areas with a total population of 579,281 persons as of 2019, the year selected for the synthetic population pipeline (see Fig. 7). Table 2 summaries demographic data for the Gothenburg region from the SCB portal:

**Table 2:** Summary of neighbourhood data.

Category	Description
Age group, gender, and household type	Demographic data on the distribution of age groups, gender, and types of households in each neighbourhood.
Household size and number of children	Data on the household size and the presence of younger and older children in households.
Cars in the neighbourhood	Statistics on total car ownership in each neighbourhood. (Note: The statistics include leased cars but not company cars)
Total workers in the neighbourhood	Data on the working population in each neighbourhood contributes to capturing economic activity and commuting patterns.
Household type data	Data on the types of houses, such as independent houses or apartment buildings, provide a view of the living arrangements in neighbourhoods.
Population data	Comprehensive population counts for residents in each neighbourhood by gender, age, household type, and number of children.
Municipal children's data	Data on households with children categorised by household type and number of children.

### 4.3 Lantmäteriet Building Footprints

The Swedish cadastral agency Lantmäteriet [5] provides a dataset of all the buildings in Sweden. We extract a subset of this dataset for Gothenburg and a LiDAR point-cloud dataset. The LiDAR point cloud is clipped to the building footprints, and the median z value is extracted. This median z value is assigned to each building as the building height. Once the building height is available, we assume a typical floor height of 3 m and calculate the number of floors in each building. The number of floors is then used to calculate the population per floor and the total feasible population for each building.

### 4.4 Transport Network

We use the osmnx [12] python package to download the street network of the Gothenburg region from OpenStreetMaps [10] as a graph object for three modes of transport: driving, pedestrian and cycling. The graph object is then converted to an iGraph [13] graph object and stored in the list of available networks. We use the OpenStreetMaps maximum speed for the driving network to determine travel time along the street edges. For the pedestrian and cycle networks, we impute the elevation at the various nodes of the street network to calculate a gradient along the street edges. The gradient then calculates the travel time along the street edges to account for impedance.

We use the r5py [14] python library to create a travel-time matrix for all OD pairs for Gothenburg at a 250-meter grid resolution and use this to query travel times when the assigned mode is public transport.

### 4.5 Gothenburg Location of Schools and Playgrounds

The city of Gothenburg [8] maintains a database of different daycare centres, pre-schools, schools, and playgrounds administered by the city. The synthetic population pipeline fetches the locations of the amenities and stores them in the list of available amenities in real time.

### 4.6 Synthetic Population Generation

The synthetic population generation consists of four sections: initialising a population, assigning origins and destinations, assigning activity sequences, and finally, performing the routing.

### 4.7 Initialising the Synthetic Population

First, we initialise a primitive synthetic population for a neighbourhood with three attributes: age, sex, and household status based on SCB data for the total population in a selected year. We choose 2019 as the simulation year to match the data available for the building footprints. Since we have access to a three-dimensional distribution, commonly used methods for synthetic population generation, such as Iterative Proportional Fitting (IPF), are unnecessary.

Table 3 outlines the household statuses considered in our synthetic population model, including their Swedish terms.

**Table 3:** Household status categories during the first initialisation of the synthetic population.

Household Status	Swedish Term	Description
Cohabiting	*Personer i samboförhållande*	A person living with a partner
Married	*Person i gift par/registrerat partnerskap*	A person in a marriage or registered partnership
Living Alone	*Ensamboende*	Individual living by themselves
Single Parent	*Ensamstående förälder*	Single adult with child(ren)
Single	*Ensamstående*	Individuals not in a marital or cohabiting relationship
Child	*Barn*	A person classified as a child in the household
Other	*Ovrigä*	Other forms of household status

### 4.8 Generating Households

**Fig. 8:**
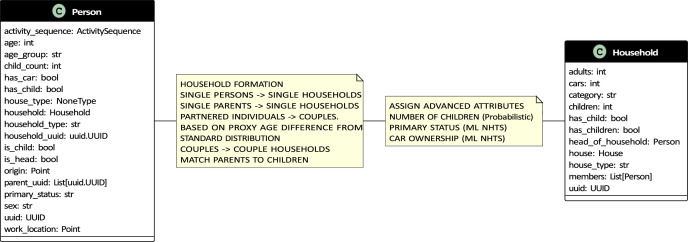
Household formation from persons.

We start by determining the total number of households in the neighbourhood. Then, we assign households to persons living alone, single parents, and cohabiting and married persons as couples. Based on the remaining number of households, we generate other households and randomly increment the number of members of the “Other” households (see Fig. 8).

### 4.9 Matching Couple Households

**Fig. 9:**
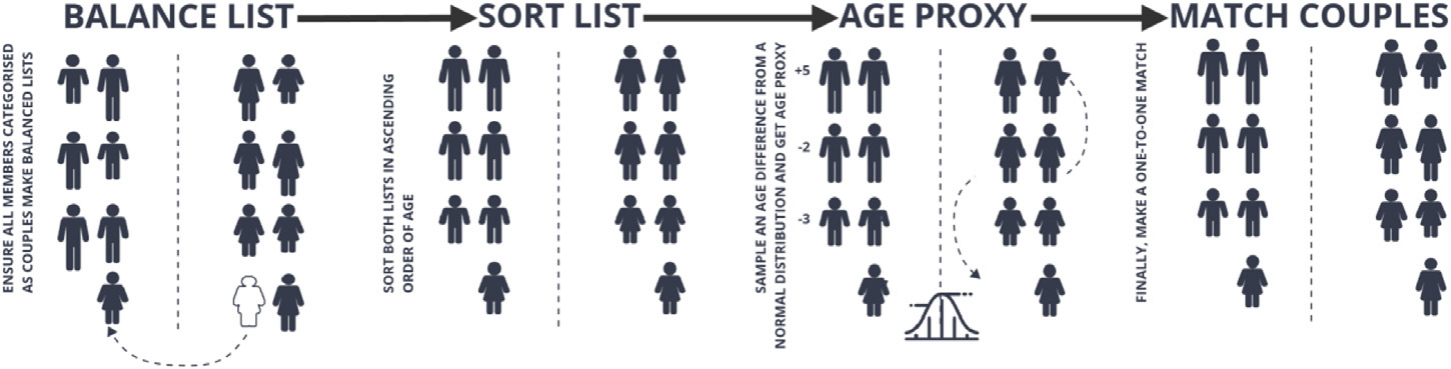
Age matching process for creating couples.

We use an age proxy heuristic for the couple matching as described in [3]. The age proxy heuristic aims to introduce variability into the age-matching process for creating couples rather than just pairing the oldest persons in one list with the oldest in another, which would be a straightforward sorting. The goal is to allow for a more natural pairing, as in real-world scenarios, age differences between couples can vary, as illustrated in Fig. 9.

1. **Divide and balance unsorted lists:** First, we create a list of males and females with a household type of *couple* as *p1* and *p2*. If the lists of males and females are not the same length, we move individuals from the longer list to the shorter one until they are the same. The individuals who will be moved between lists are chosen randomly from the longer list. Couples where partners live in different households are not considered in our model.

2. **Sort lists and create an age proxy:** Each person *i* in list *p1* calculates a proxy_age*_i_* by adding their actual age (age*_i_*) to a number *N*(0*,*6) sampled from a normal distribution with mean 0 and standard deviation 6:

(1) $$\text{proxy}\_\text{ag}e_{i}=\text{ag}e_{i}+N\left(0,6\right)$$

3. **Sorting by age proxy:** In descending order, the individuals in list p1 are sorted by their proxy ages. Such that, if we have proxy ages proxy_age_1_*,* proxy_age_2_*...,* proxy_age*_n_*, we reorder p1 so that:

(2) $$\text{proxy}\_\text{ag}e_{\pi \left(1\right)}\geq \text{proxy}\text{ag}e_{\pi \left(2\right)}\geq \ldots \geq \text{proxy}\text{ag}e_{\pi \left(n\right)}$$

where *π* is a permutation of the indices 1 through *n* that sorts *p1* by the proxy ages.

4. **Match couples and form households:** After sorting, individuals from *p2* and *p2* are paired by their indices. Therefore, the first person in the sorted p1 list is paired with the first person in *p2*. This pairing allows for variability in age differences because the proxy ages introduce randomness into the sort order of *p1*.

A household is created for each pair, and the two individuals become members of that household. If the individual from *p1* is older than the one from *p2*, they are designated as the head of the household.

### 4.10 Children Assignment

We use a similar technique for assigning children to households. Let *C* represent the set of all children who require assignment to households, and *H* denote the set of all households. Within set *H*, a subset *H_c_* exists that must receive children. If *h* is a household within *H*, the capacity of each household *h* to accommodate children is determined by the function *cap*(*h*), which returns the maximum number of children a household can accommodate based on a distribution of the number of children per household at each neighbourhood level. The children's assignment is done in three steps, subject to two constraints. First, the capacity constraint ensures that the number of children allocated to any given household does not surpass its maximum capacity - for every household *h* within the set of considered households *H_c_*, the total count of children *count*(*h*) assigned to *h* must be less than or equal to the household's capacity *cap*(*h*). The second constraint relates to the age of the household head, such that the household head must meet or exceed a specified minimum age threshold to qualify for child assignment. For each household *h* in *H_c_*, the age of the household head *age_head_*(*h*) should be greater than or equal to the minimum required age *age_head_*_min_.

The steps are as follows:

1. **Household Selection**: Each household *h*∈*H* can have zero or more children up to its capacity cap(*h*). This heuristic defines a set *H_c_* ⊆ *H* where households have at least one child.
2. **Assignment Function**: The assignment function assign (*c, h*) maps children to households. For every child *c*∈$C^{\prime }$, there exists a household *h*∈*H* such that *c* is assigned to *h*, which can be expressed as:
  (3) $$\forall c\in c^{\prime },\exists \;h\in H|\text{assign}\left(c,h\right)$$

Given that the household *h* has not yet reached its capacity cap(*h*).

3. **Distribution of Children**: Children (*c_i_*) are distributed among households (*h_j_*) in a way that fills each household before assigning children to the next household. This distribution can be expressed by an ordered assignment process, where households are filled sequentially:

(4) $$\text{assign}\left(c_{i},h_{j}\right)\to \text{assign}\left(c_{i+1},h_{j}\right)\;\text{if}\;\text{cap}\left(h_{j}\right)>\text{count}\left(h_{j}\right)$$

(5) $$\text{assign}\left(c_{i},h_{j+1}\right)\;\text{if}\;\text{cap}\left(h_{j}\right)=\text{count}\left(h_{j}\right)$$

where count(*h_j_*) is the number of children $c_{i}$ currently assigned to household *h_j_*.

### 4.11 Machine Learning-Based Attributes

**Fig. 10:**
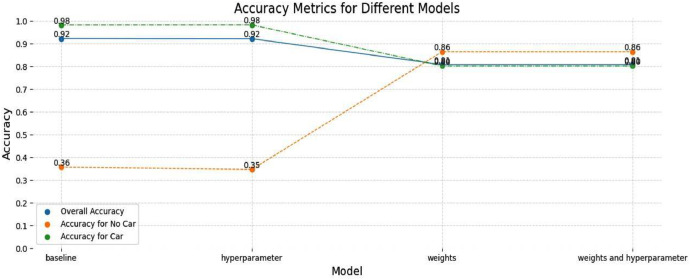
Performance of different ML models in predicting car ownership.

We use a machine learning (ML) model to determine the number of cars in a household and another ML model to determine the primary status of each person above 18. We begin by performing exploratory analysis on the different variables in the NHTS dataset and select the ones with high feature importance in predicting the number of cars and primary status. We found similar variables as described in Hubert and Toint [15], Avery [16] and Cornelis et al. [17], such as age, sex, household type, house type and number of children in the household. Next, we define car ownership as a binary category where the model must predict whether a person has a car (or not). For the primary status, categories for classification are divided between working, studying and neither. For both models mentioned above, we perform one-hot encoding on the categorical variables and prepare the data for ML. We used a random forest classifier and explored different data augmentation steps like hyperparameter tuning and synthetic minority oversampling techniques to deal with the imbalance in the dataset (see Fig. 10). We then use the trained model to predict the number of cars in each household and the primary status of each person above 18.

### 4.12 Assigning Origins and Destinations

Each household is assigned a house object that can be accommodated into a building object. A house object is automatically created when a household is assigned to a building.

### 4.13 Assigning Homes and Buildings

The first step in assigning households to a building is to pre-process all the buildings in the neighbourhood. To do this, we filter all buildings from the building footprint data by attributes associated with residential buildings. Then, we filter buildings smaller than the average area per person for Gothenburg, 36 m^2^s [6]. Next, the total built-up area is calculated and refactored only to include liveable areas; we do this by assigning a service area factor of 0.15 for multi-family houses and 0.09 for single-family houses. These values are estimated based on reference floor plans of houses in Gothenburg. Finally, we estimate the population per floor of all buildings.

Next, we split the list of households into single-family and multi-family households and sorted them by the number of members from largest to smallest. Similarly, we also group, split, and sort the buildings.

Single-family households are assigned to buildings by iterating over the list of single-family households and assigning them to the first building with enough space. The process is repeated until all single-family households are assigned to a building.

The allocation process for multi-family households involves distributing them among the available multi-family buildings. This allocation is done by calculating the total number of multi-family households and the total number of multi-family buildings available. The households are then assigned to buildings in a manner that aims to distribute the population evenly, taking into consideration the capacity of each building.

A cycle-based allocation system is used to achieve this distribution. Initially, the number of cycles is determined by dividing the total number of multi-family households by the total number of multi-family buildings, ensuring each building receives an approximately equal number of households. In each cycle, households are sequentially assigned to buildings, with the condition that a building can only accommodate a new household if it has sufficient remaining capacity. The capacity is calculated based on the area per person criterion and the total built-up area of the building minus the population already accommodated in the building. Suppose a building reaches its capacity during the allocation process; it is skipped for the remainder of the current cycle, and the next household is considered for the subsequent building with available capacity. This process continues until all households are assigned or all buildings have reached capacity.

After completing the primary allocation cycles, a set of households may remain unallocated due to the division remainder. These households are distributed in a final round, following the same capacity constraints, starting from the first building in the list and continuing sequentially until all remaining households are accommodated.

The building allocation process can be represented as follows:

Let *H* = {*h*_1_*, h*_2…_ *h_nh_*} be the set of multi-family households and *B* = {*b*_1_*, b*_2…_*,b_nb_*} be the set of multi-family buildings. Each household *h_i_* has a certain number of members *m_i_*, and each building *b_j_* has a built-up area *A_j_* and an existing population *P_j_*. The area required per person is denoted as *a*.

1. Calculate the total number of cycles, *C*, as:

(6) $$C=\lfloor \frac{n_{h}}{n_{b}}$$

2. For each cycle *c* from 1 to *C*, and each building *b_j_* in *B*:

•Assign a household *h_i_* to *b_j_* if the remaining capacity of *b_j_* is sufficient. The remaining capacity *R_j_* of *b_j_* is given by:

(7) $$R_{j}=a\cdot A_{j}-P_{j}$$

•If the remaining capacity *R_j_* is greater than or equal to the number of members *m_i_* in *h_i_*, assign *h_i_* to *b_j_* and update *P_j_*:

(8) Pj=Pj+mi

•If *R_j_* is less than *m_i_*, proceed to the next building.

3. For the remaining households, distribute them starting from the first building in *B* and following the same capacity check as above.

### 4.14 Computing Preferred Locations

**Fig. 11:**
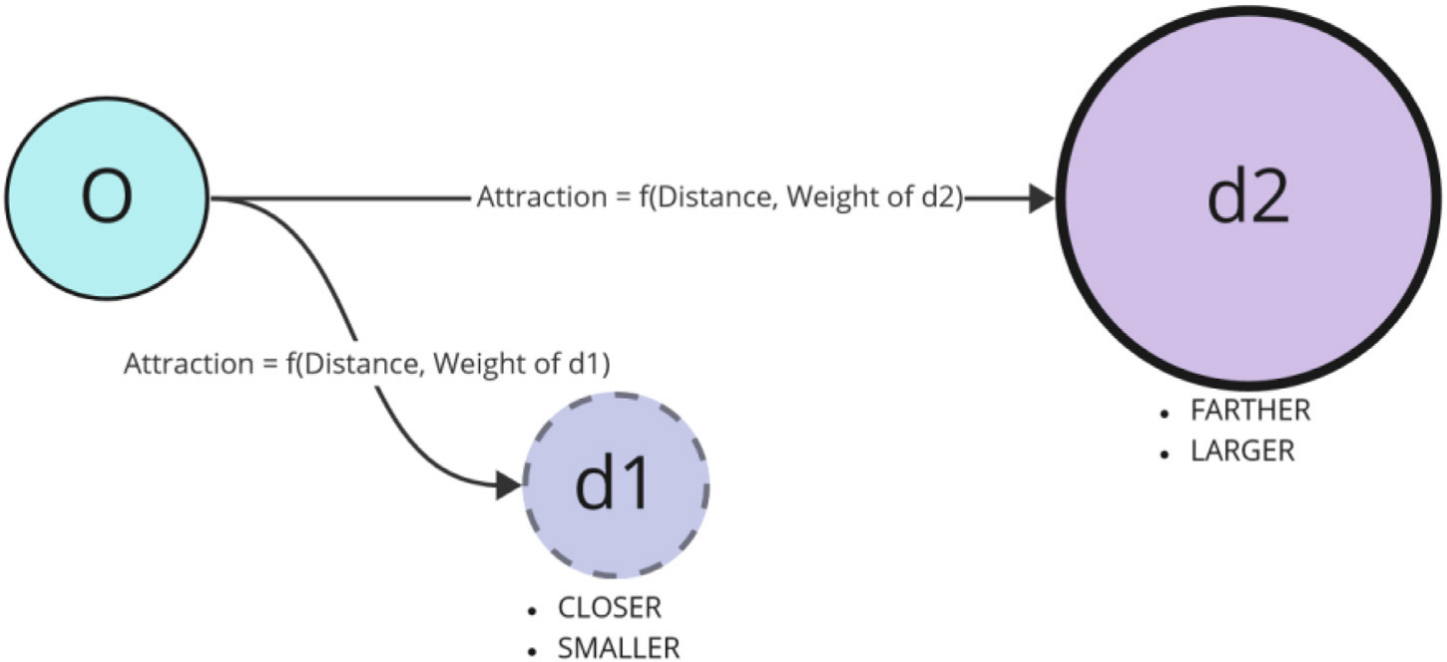
Illustration for gravity model.

The gravity model used in the context of finding the optimal destinations, such as a grocery store location, is a conceptual tool based on the law of universal gravitation in physics, which states that every point's mass attracts every other point's mass with a force that is directly proportional to the product of their masses and inversely proportional to the square of the distance between their centres. In spatial and urban analysis, the gravity model estimates the ”attraction” between locations based on size and distance (See Fig. 11).

For grocery stores, the gravity model is implemented as follows:

1. **Area as mass**: The area of the grocery store is analogous to the mass in the gravitational equation. A larger grocery store (larger area) is assumed to have a greater” pull” or attraction because it can offer more products and services and generally more convenience to the customer.
2. **Distance as separation**: The distance between the customer's location (origin point) and the grocery store represents the separation between two masses in the gravitational equation. The farther a customer is from a store, the less likely they are to be” attracted” to it because the inconvenience of travelling increases with distance.
3. **Weighting with coefficients**: The coefficients *α* and *β* are exponents for the area and distance, respectively, to weigh their importance in the gravity model. The value of *α* emphasises the importance of store size (area) in the attraction, while *β* controls how quickly the attraction decreases with distance (distance decay). These coefficients can be adjusted based on empirical data or policy goals.

The gravity score formula derived from this model for a grocery store at a distance *d* from a customer and with area *A* is:

(9) $$\text{Gravity}\;\text{score}=\frac{A^{\alpha }}{{\left(d+\epsilon \right)}^{\beta }}$$

Where:

• *A* is the area of the grocery store.
• *d* is the distance between the customer's location and the grocery store.
• *α* is the exponent for the area, indicating its relative importance.
• *β* is the exponent for distance, representing the distance decay effect.
• *ϵ* is a small constant to prevent division by zero, ensuring that the formula is well-defined even when *d* is minimal.

According to the gravity model, the optimal grocery store has the highest gravity score when considering a particular origin point. This store would theoretically have the best combination of being large enough to attract customers (high area score) and close enough to minimise inconvenience (low distance score). In practice, one would calculate the gravity score for all accessible grocery stores from the origin point and select the store with the highest score as the optimal choice.

For other amenities like healthcare, leisure and education, the model selects the closest location that satisfies the requirements.

### 4.15 Computing Work Locations

Again, we use the gravity model to assign workers a job location. The process involves filtering buildings to determine potential job locations, computing job densities for these locations, using the gravity model to assess the attractiveness of job locations for each home and finally assigning jobs to workers in each house. The process can be tuned using the coefficients in the gravity model (see Fig. 2).

The following steps are used to determine job locations on an individual basis:

1. Filtering buildings and calculating the total built-up area

Given the initial set of buildings *B*, we filter residential buildings and compute the Gross Floor Area (GFA).

(10) $$B^{\prime }=\left\{b\in B∥\text{function}\left(b\right)\in \left\{{}^{\prime \prime }residential{}^{\prime \prime },\text{“}unspecified^{\prime \prime }\right\}\wedge \text{area}\left(b\right)>500\right\}$$

Where function(*b*) returns the function of building *b* and area(*b*) returns the area of building *b*.

For each building $b^{\prime }$ in $B^{\prime }$, the GFA is computed as:

(11) $$\text{GFA}\left(b^{\prime }\right)=\text{area}\left(b^{\prime }\right)\times \text{height}\left(b^{\prime }\right)$$

2. Scaling total jobs to compute jobs in a 1000 m radius

The number of jobs per square meter *J_m_* is computed as:

(12) $$J_{m}=\frac{T}{\sum_{b^{\prime }\in B^{\prime }}\text{GFA}\left(b^{\prime }\right)}$$

Where *T* is the total number of workers in Gothenburg and $b^{\prime }$ is a building belonging to the set of all buildings $B^{\prime }$ within a unit region.

3. Job density calculation

For each job location *j*, its job density *D*(*j*) is determined based on other job locations within a given radius *r*:

(13) $$D\left(j\right)=\frac{\sum_{j^{\prime }\in N\left(j,r\right)}\text{jobs}\left(j^{\prime }\right)}{\pi r^{2}}$$

*N* (*j, r*) is the set of job locations within radius *r* of job location *j,* and jobs($j^{\prime }$) is the number of jobs at location $j^{\prime }$.

4. Gravity model to find suitable jobs

The interaction *I* between two places, say a home *h* and a job location *j*, is determined using the gravity model:

(14) $$I\left(h,j\right)=\frac{P\left(h\right)\times P\left(j\right)\times D{\left(j\right)}^{w_{D}}}{d{\left(h,j\right)}^{w_{DD}}}$$

Where:

• *P*(*h*) is the population (or number of workers) at home location *h*.
• *P*(*j*) is the number of jobs at job location *j*.
• *D*(*j*) is the job density at job location *j*.
• *d* (*h, j*) is the distance between home *h* and job location *j*.
• *w_D_* and *w_DD_* are the density and distance decay weights, respectively.

For each home *h*, the top *N* job locations are selected based on the highest values of *I* (*h, j*).

5. Job assignment

Given that each home *h* has *n* workers and a list of potential job locations based on the gravity model, the jobs are assigned as follows:

For each worker in *h*:

• Assign one of the potential job locations.
• Update the list of potential job locations (by either removing the assigned location or adjusting it as per other criteria).

6. Tuning with coefficients

The coefficients *w_D_* and *w_DD_* in the gravity model act as parameters that can adjust the significance of job density and distance in determining interactions. By adjusting these coefficients:

• Increasing *w_D_* will give more significance to job density.
• Increasing *w_DD_* will give more significance to distance, making closer locations more attractive.

### 4.16 Sampling Activity Sequences

In previous steps, a person has a primary activity assigned to them through the ML model. Based on the demographic attributes of the person and the two predicted attributes of primary status and car ownership, we perform a statistical matching of activity sequence from the NHTS based on D'Orazio et al. [18]; Namazi-Rad et al. [19] (See Fig. 4).

However, the NHTS samples must be validated to ensure they are valid sequences. This validation process includes checking for null sequences, sequences with negative or zero duration, duration that extends over 24 h, and an activity performed after every travel activity. After performing the validation, we see that roughly 12 % of sampled activity chains fail the validation.

### 4.17 Computing Routes

Routing is the process of finding the optimal path between two points in a network, such as a road network. For active mobility, routing algorithms can be used to find the best walking or cycling route between two locations, considering factors such as distance, slope, and the presence of natural features through weighting the edges of the network. This weighting can help residents find efficient travel time and effort routes that are pleasant to use. The following variables are used to generate an edge weight to select the optimal route.

• **Length (L)**: Shorter distances are generally preferred for active mobility, as they reduce travel time and physical exertion.
• **Nature Gradient (N)**: Routes closer to nature can be more aesthetically pleasing and offer a more enjoyable experience, which is particularly important for active mobility users like walkers and cyclists.
• **Slope (S)**: Flatter routes are usually preferred as they require less energy, making them more accessible and comfortable for users, especially those with mobility issues or riding bicycles.

The routing algorithm thus models the real-world preference for shorter, flatter, and more scenic routes, providing a balanced path that considers distance, effort, and environmental quality.

In addition to active mobility modes like walking and cycling, routing algorithms can also be applied to public transportation networks to find the best bus or train route between two locations using the r5py [14] library and the regional GTFS data [11].

Finally, we perform routing on car trips using the travel time as the edge weight using iGraph by Csardi and Nepusz [13] and the Dijkstra algorithm.

### 4.18 Validation

The synthetic population generation consisted of four sections: initialising a population, assigning origins and destinations, assigning activity sequences, and performing the routing. We first looked at disjointed and joint distributions of different variables to evaluate the synthetic population. Then, we looked at the routing and mobility results to evaluate whether the model successfully captured the nuances of the built environment and demographic changes and recreated a local activity demand sequence and activity engagement profile.

### 4.19 Disjointed Variables

While the model samples from a three-dimensional distribution of demographic characteristics, constraints on household sizes and couple matching in the pipeline can cause inconsistencies in these attributes due to errors in the data or how certain variables are defined. One such example is the definition of a child. In one statistic, a child may be an individual who lives in the same household as their parent regardless of age. In contrast, another statistic may define a child as an individual under 18.

**Fig. 12:**
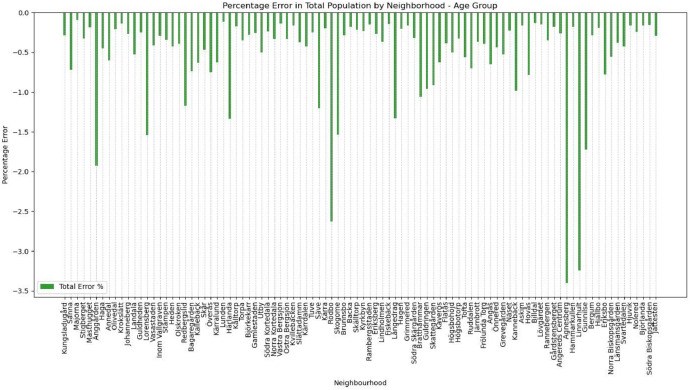
Percentage error of the age group distribution in the synthetic population compared to the actual population.

**Fig. 13:**
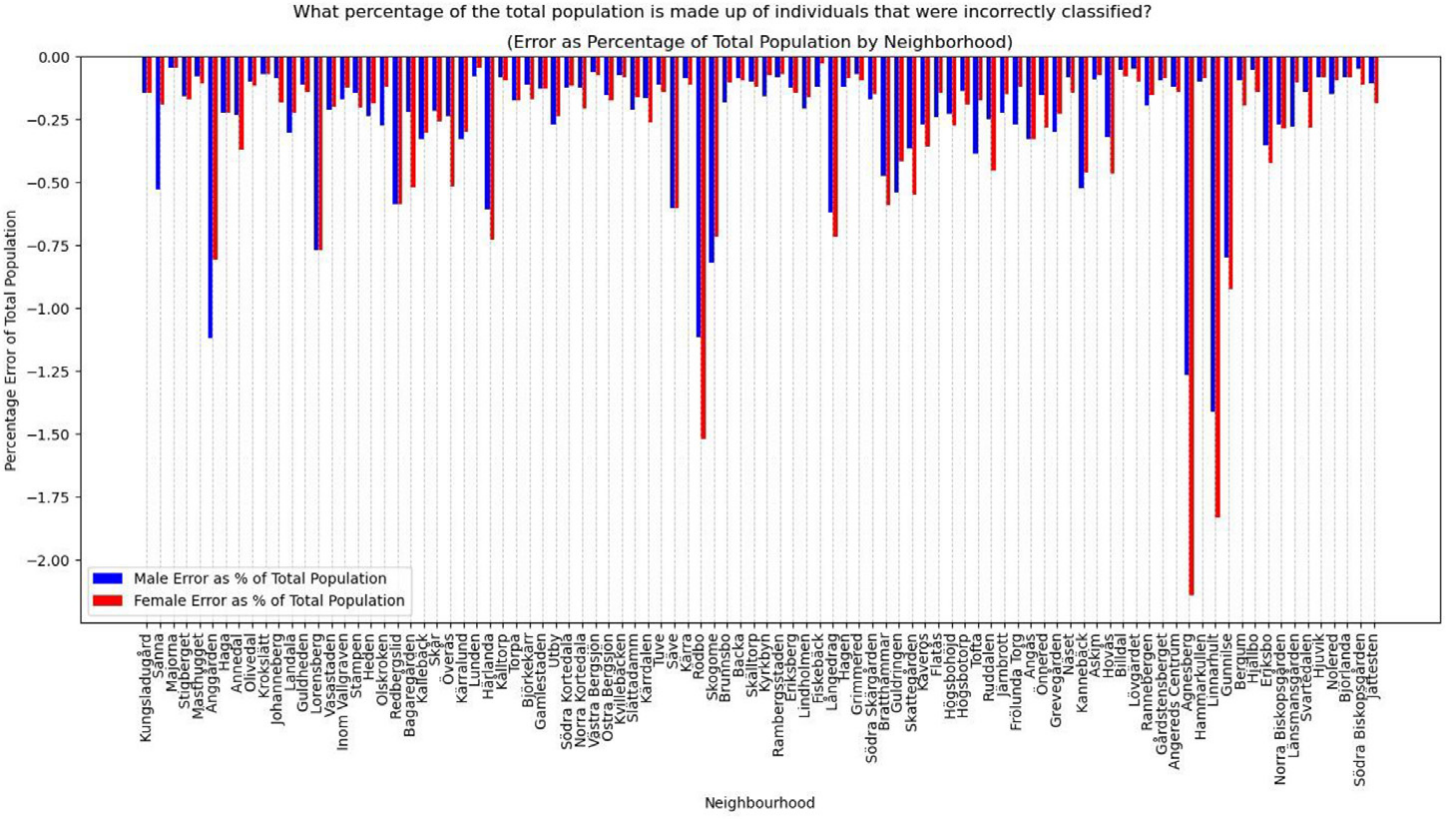
Percentage error of the sex distribution in the synthetic population compared to the actual population.

To evaluate the fundamental attributes, we first look at disjointed variables of age group and sex (see Figs. 12 and 13)

**Fig. 14:**
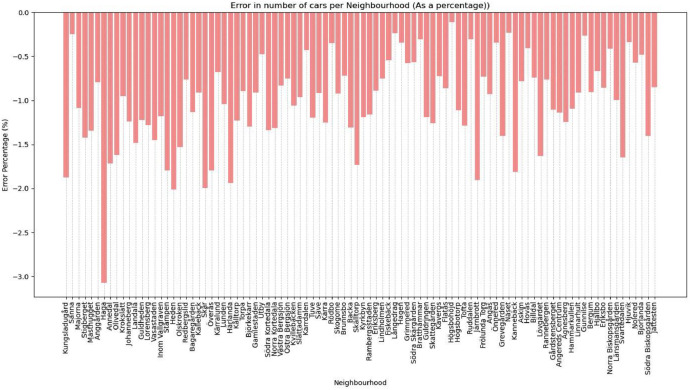
Percentage error of car ownership in the synthetic population compared to the actual population.

Then, we look at disjointed variables for the predicted variables. While the ML model was validated to have relatively high accuracy nationally, we need to ensure that locally imputing these national-level attributes can reliably reproduce the numbers for car ownership (see Fig. 14).

The evaluation shows that of the 95 residential neighbourhoods (one of the Gothenburg neighbourhoods, Arendal, is omitted as an industrial area), 94 are below a 2 % error percentage. Effectively, <2 % of the synthetic population is incorrectly classified in the disjointed attributes.

### 4.20 Joint Distributions

**Fig. 15:**
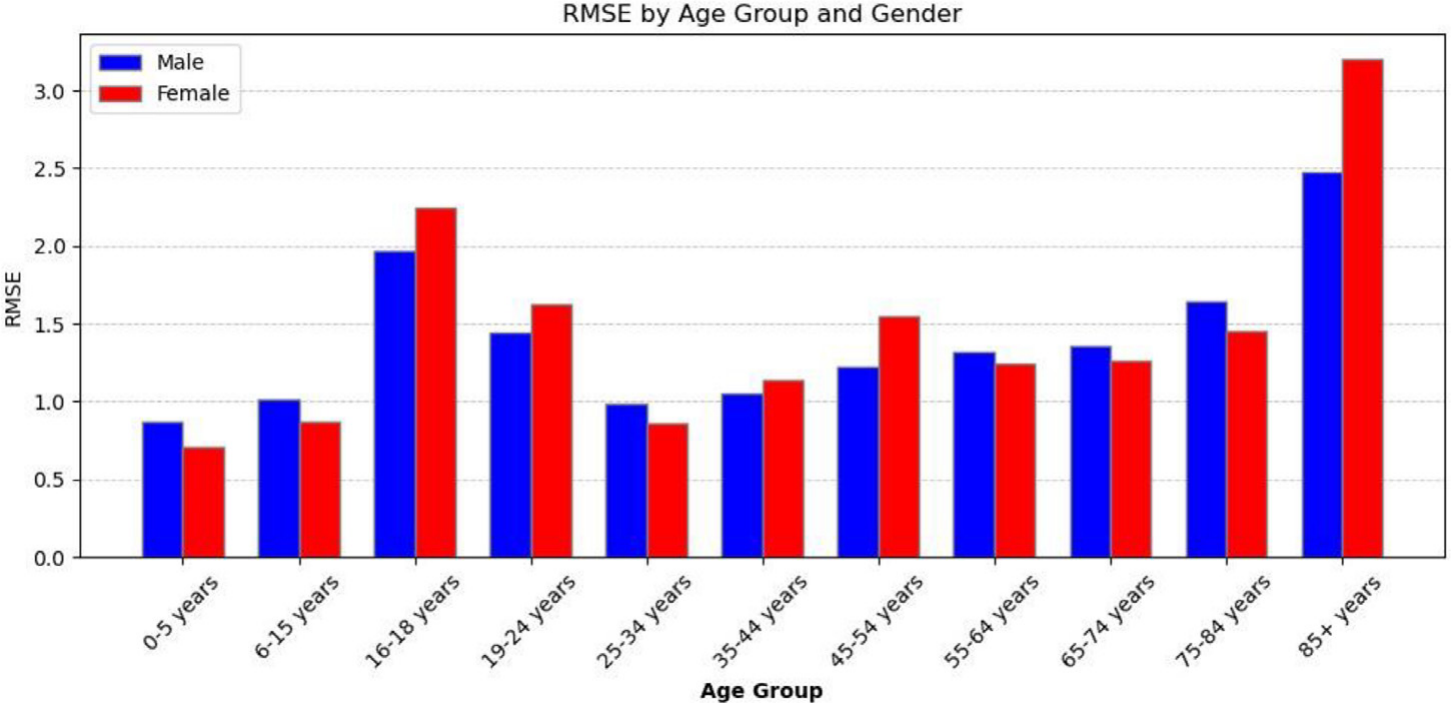
RMSE of the joint distribution of age and sex in the synthetic population compared to the actual population.

Next, we look at the accuracy of the joint distributions. Here, we look at the root of the mean squared error at the intersection of variables for age group and sex (see Fig. 15).

We see an average RMSE of 2–3 per neighbourhood, with older age groups being underrepresented. This underrepresentation is due to the sorting by age step in the formation of households.

### 4.21 Routing and Mobility

**Fig. 16:**
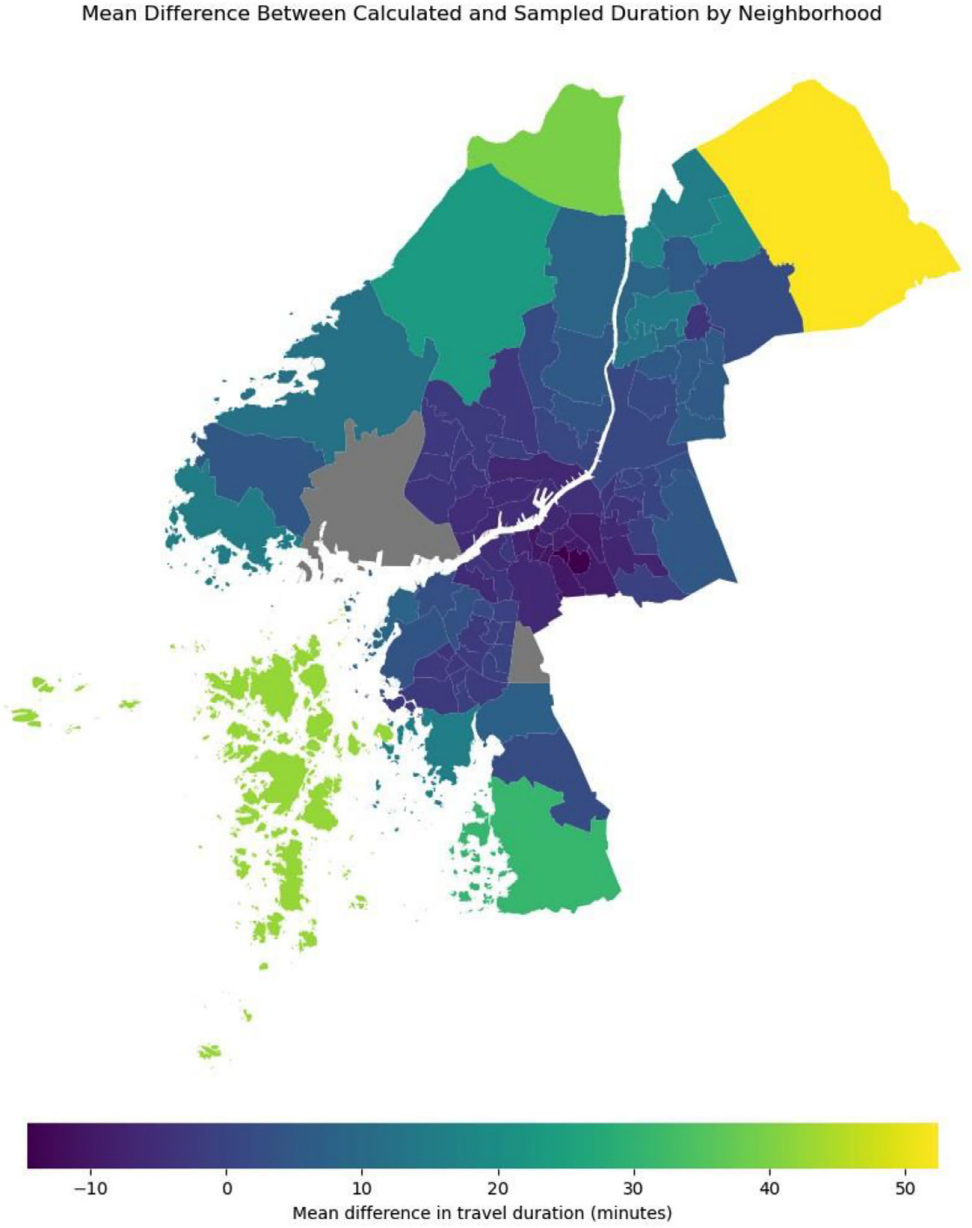
Difference in simulated travel time in Gothenburg compared to NHTS.

Finally, the model was developed to impute activity demand and mobility behaviour from a national scale through the NHTS to a neighbourhood scale. Fig. 16 compares the average travel time in each neighbourhood of Gothenburg to the mean travel time from the NHTS. We can see that more centrally located neighbourhoods with a higher density of amenities report lower than the average NHTS travel time, and suburban neighbourhoods with a lower density of amenities report higher than the NHTS mean travel time.

While Fig. 16 shows aggregated and averaged travel times, the model generates a temporally and spatially disaggregated synthetic dataset. For example, in Fig. 5, Fig. 6, we can see temporally aggregated metrics for each neighbourhood.

### 4.22 Handling High Divergence

The validation process revealed some divergence between the synthetic and actual populations. Specifically, the RMSE for age groups is less than three years, the percentage error for car ownership is <3 %, and the percentage of residents incorrectly classified in their gender attributes is <2 % at a neighbourhood level. These divergences reflect the inherent challenges in replicating real-world demographics and travel behaviours. Depending on the specific application of the model, these errors could be further reduced in the following iterations of the model through the following:

• Incorporating additional socio-economic variables like median income.
• Improving the accuracy of the ML models by employing ensemble learning models rather than making binary classifications.
• Updating the model with more recent and comprehensive data.

For the routing component of the model, future iterations could benefit from enhancements in:

• Enriching the destination database with additional amenity attributes.
• Including mobility heuristics derived from data using actual GPS traces.
• Including representative travel speeds for active mobility depending on residents’ age.

While there are divergences in the current dataset generated from the model, it is designed with the flexibility to incorporate future improvements based on data availability and intended use case. These refinements would address the observed divergence and enhance the models’ applicability across application domains.

## Limitations

For the synthetic population generation, the economic attributes of individuals or households are not directly accounted for in the model. Instead, we impute this through the car ownership data, which serves as a proxy for the economic status of an individual. This modelling heuristic is a limitation of the model and may affect the results depending on the application of the data. Additional variables like income and education could be added to improve the model's accuracy. While this would require additional data collection, the model framework is flexible enough to allow for the addition of such variables.

The couple formation method uses two lists of male and female partners. Same-sex couples are only formed during the re-balancing of the lists, which is an extreme oversimplification of real-world couple formation. This limitation of the model must be considered when interpreting the results of the model depending on the application of the data.

Another limitation of the model is that it uses statistical matching to derive activity sequences from an NHTS. As a result, individuals under 18 do not have an activity sequence. The model has additional mechanisms that allow procedurally generating activity sequences of minors, but this is not included in the current dataset. Compared to the state-of-the-art methods in synthetic population generation, our model does not use a logit-based choice mechanism to allow each resident to “choose” their mode of transport based on the destination. Instead, the mode choice and activity sequence are sampled from the NHTS.

Finally, for the public transportation mode of travel, we use a peak hour departure window between 8 am and 10 am to derive the travel time matrix. Therefore, the travel times using public transport overestimate how long residents need to travel to their destinations.

## Ethics Statement

The authors have received permission from the respective authorities to use the Swedish NHTS and building footprints. All other data sources used in this research are from open data sources. All data employed in this study were fully anonymised before analysis, ensuring no personally identifiable information was utilised. The dataset comprises synthesised individuals and households created using open demographic data without directly referencing specific real-world individuals.

## Declaration of Generative AI and AI-Assisted Technologies in the Writing Process

During the preparation of this work, the author(s) used chatGPT to improve the manuscript's readability and prepare latex formulas. After using this tool/service, the author(s) reviewed and edited the content as needed and take(s) full responsibility for the publication's content.

## CRediT authorship contribution statement

**Sanjay Somanath:** Conceptualization, Methodology, Software, Writing – original draft, Validation. **Liane Thuvander:** Writing – review & editing, Supervision. **Alexander Hollberg:** Writing – review & editing, Supervision.

## Data Availability

Activity based synthetic population of residents for Gothenburg, Sweden (Original data) (Zenodo).
